# Access to primary health care services for Indigenous peoples: A framework synthesis

**DOI:** 10.1186/s12939-016-0450-5

**Published:** 2016-09-30

**Authors:** Carol Davy, Stephen Harfield, Alexa McArthur, Zachary Munn, Alex Brown

**Affiliations:** 1 Wardliparingga Aboriginal Research Unit, South Australian Health and Medical Research Institute, Adelaide, SA 5000 Australia; 2 Joanna Briggs Institute, University of Adelaide, Adelaide, SA 5000 Australia

**Keywords:** Indigenous, Aboriginal, First Nation, Maori, Primary health care, Models of service delivery

## Abstract

**Background:**

Indigenous peoples often find it difficult to access appropriate mainstream primary health care services. Securing access to primary health care services requires more than just services that are situated within easy reach. Ensuring the accessibility of health care for Indigenous peoples who are often faced with a vast array of additional barriers including experiences of discrimination and racism, can be complex. This framework synthesis aimed to identify issues that hindered Indigenous peoples from accessing primary health care and then explore how, if at all, these were addressed by Indigenous health care services.

**Methods:**

To be included in this framework synthesis papers must have presented findings focused on access to (factors relating to Indigenous peoples, their families and their communities) or accessibility of Indigenous primary health care services. Findings were imported into NVivo and a framework analysis undertaken whereby findings were coded to and then thematically analysed using Levesque and colleague’s accessibility framework.

**Results:**

Issues relating to the cultural and social determinants of health such as unemployment and low levels of education influenced whether Indigenous patients, their families and communities were able to access health care. Indigenous health care services addressed these issues in a number of ways including the provision of transport to and from appointments, a reduction in health care costs for people on low incomes and close consultation with, if not the direct involvement of, community members in identifying and then addressing health care needs.

**Conclusions:**

Indigenous health care services appear to be best placed to overcome both the social and cultural determinants of health which hamper Indigenous peoples from accessing health care. Findings of this synthesis also suggest that Levesque and colleague’s accessibility framework should be broadened to include factors related to the health care system such as funding.

**Electronic supplementary material:**

The online version of this article (doi:10.1186/s12939-016-0450-5) contains supplementary material, which is available to authorized users.

## Background

Ensuring access to primary health care is widely accepted as key to improving health outcomes [1]. In the case of Indigenous populations living with high rates of chronic disease, access to these services is even more crucial [2]. Even in developed countries such as Australia, the number of Indigenous peoples dying from cardiovascular disease is 1.5 times that of their non-Indigenous counterparts [3]. Despite this, Indigenous peoples are often prevented from accessing these types of services due to a range of barriers including the high cost of health care, experiences of discrimination and racism and poor communication with health care professionals [4]. Evidence suggests that access to primary health care can be improved when services are tailored to the needs of, or owned and managed by Indigenous communities themselves [5, 6]. This is because Indigenous health care services are more likely to be free of racism and are generally more culturally appropriate than mainstream services [7]. They also tend to employ Indigenous staff who are able to speak the local language and are often known by people accessing the service [8].

Accessing primary health care is therefore far more complex than simply locating a service within or close to Indigenous communities [9]. Nevertheless, measures of access at a population level are often confined to spatial factors including location and distance, using primarily quantitative data. In Australia, for example, a rural index of access combines system measures such as the number of health services within a given area and the population-provider ratio, with measures including the type and degree of identified health needs, distance to the nearest service and a mobility score [10]. Others have used less complex scores focusing on distance [11], travel time [12] and supply and demand ratios [13]. Quantitative measures of socioeconomic status with indicators of disadvantage have also been included [14, 15]. Clearly these quantitative perspectives ignore many of the access issues relevant to Indigenous peoples such as the ability of the service to accommodate the social and cultural needs of Indigenous peoples, the provision of health care by Indigenous staff in an Indigenous friendly space and considering the important role that communities and families often play within the care process [16].

Problems associated with defining access have contributed to the complexity about what should be measured. Early research defined access in terms of how well available services are able to meet the health needs of the populations they serve [17], or alternatively how well patients are able to access health care given their particular capacity to seek and obtain care [18, 19]. These types of definitions tend to place responsibility for access on either the health service or the potential user. Not all researchers agree with this dichotomy. For example Haggerty et al. [20] suggested that both provider and patient factors are important, suggesting that the term ‘access’ best refers to the ability of the population to obtain appropriate health care services, while ‘accessibility’ best refers to the ability of the health care service or system to respond to those needs. Other frameworks have exemplified the importance of the user/service interface [21] suggesting that access to health care is jointly negotiated between the patient and the health care service [22].

**Fig. 1:**
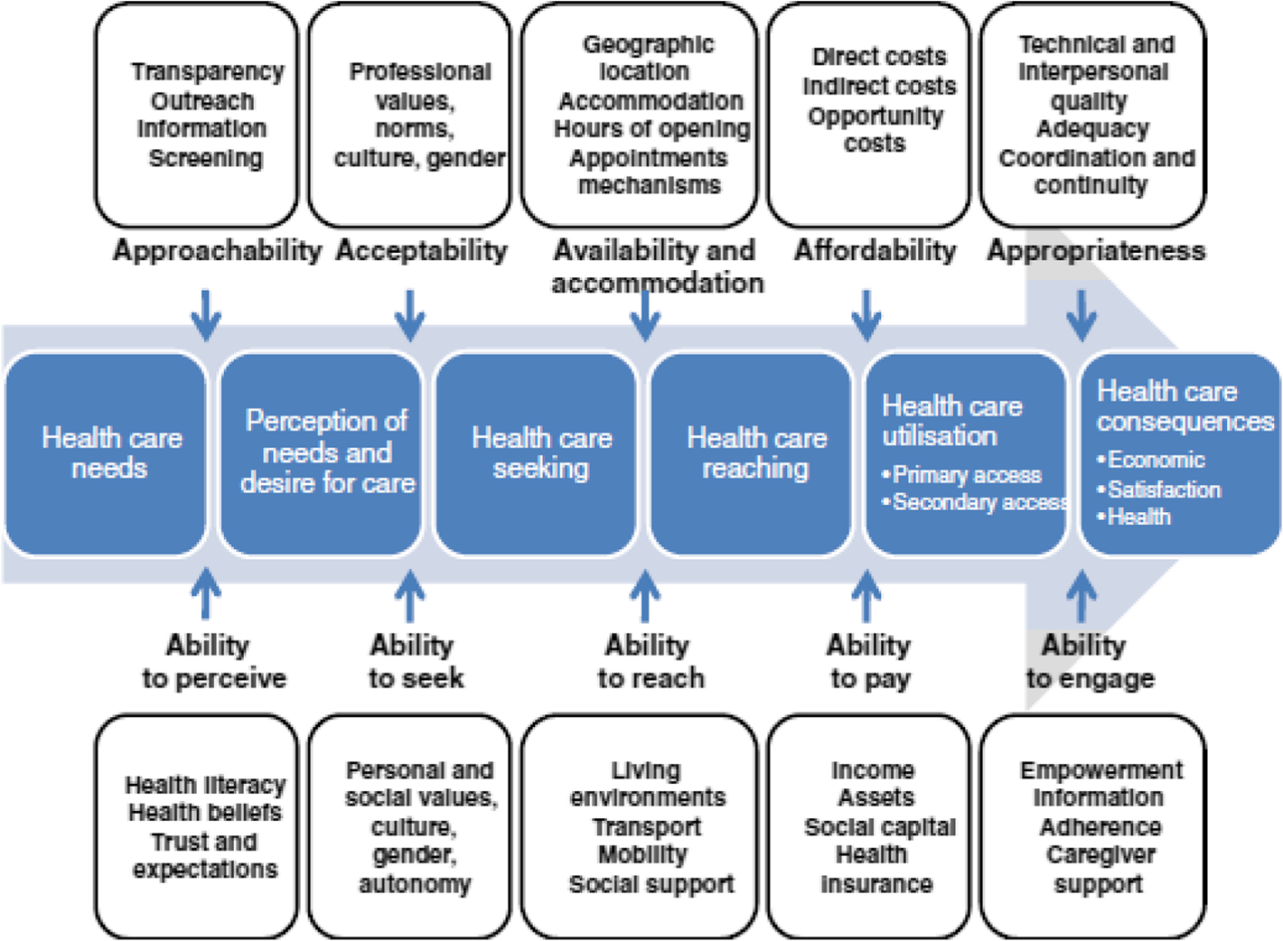
Levesque et al. [23] model of access to health care reprinted with permission

Levesque et al. [23] built upon this earlier research by developing a more holistic conceptual framework based around broader definitions of access to and accessibility of health care services. Both user and health care service characteristics are incorporated within the five stage linear framework. The strength of Levesque et al’s framework is that their model of access does not stop at the user reaching the health care service but instead considers important access issues in relation to Indigenous peoples engaging with and remaining engaged with care overtime [24]. Stage one, *Perception of Needs and Desire for Health Care*, is influenced by the ability of people to recognise a need to seek care (Ability to Perceive) and by the degree to which the health care service is known to exist (Approachability). Stage two, *Health Care Seeking*, focuses on the ability of people to freely seek out services when needed (Ability to Seek) and the appropriateness of health care services relating to, for example, the social and cultural norms that underpin the communities they serve (Acceptability). Stage three, *Health Care Reaching*, focuses on how easy it is for individuals to get to the service when needed (Ability to Reach) and whether health care services can be reached in a timely manner (Availability and Accommodation). Step four, *Health Care Utilisation*, encompasses the cost to patients accessing services (Ability to Pay) and the expenses incurred in running a health care service (Affordability). Stage five, *Consequences of Accessing Health Care*, considers how well the individual is able to engage with the care that is offered (Ability to Engage) and the extent to which the care provided meets the needs of the communities they serve (Appropriateness) (Fig. 1).

A recently completed scoping review [25] identified and described the characteristics (values, principles, components and suggested practical applications) of models of service delivery implemented within primary health care services that predominantly provide care for Indigenous people worldwide. One of the key characteristics which underpinned these models of service delivery was access. In particular, these initial findings suggested that community needed to be aware of the service and that services in turn need to ensure that they provided affordable, available and acceptable care. The primary objective of the framework synthesis presented in this paper was to systematically re-examine literature included within the previous scoping review in order to identify and better understand factors that influenced access to and accessibility of these Indigenous health services.

Our framework synthesis primarily aimed to identify the challenges faced by Indigenous peoples attempting to access care and then explore how Indigenous health care services addressed those challenges using the more holistic framework developed by Levesque et al. [23]. Identifying both the challenges and the ways in which they have been addressed will assist mainstream services to improve the accessibility of their health care. We also sought to explore whether the framework developed by Levesque and colleagues was useful for exploring access to and the accessibility of Indigenous health care services in particular. To our knowledge this is the first synthesis to look at the issues of both access and accessibility of Indigenous health care services using Levesque et al’s framework.

## Methods

The focus and design of the original scoping review and this framework synthesis were guided by a Leadership Group comprising 24 senior members of the Aboriginal Community Controlled Health Sector. The original scoping review research team, led by an Aboriginal Research Fellow was also involved in this new framework synthesis. While all of the authors were experienced in synthesising data, we believe our findings were strengthened by the involvement of Indigenous researchers who guided the interpretation of data.

### The original scoping review

The original scoping review summarised below aimed to identify the characteristics (values, principles and components) of Indigenous primary health care service delivery models. Further information on the methods used and the design for the scoping review can be found in the published protocol [25].

#### Concept

Concepts of interests included characteristics (values, principles, components and suggested practical applications) of models of service delivery implemented within an Indigenous primary health service. Within the literature a number of different terms such as service delivery models of care and service frameworks have been used interchangeably to articulate the way in which services are or should be operationalised.

#### Context

Service delivery models implemented within settings where primary health care services were provided predominantly for Indigenous peoples were included in the original scoping review. Indigenous peoples were defined as:

“Indigenous populations are communities that live within, or are attached to, geographically distinct traditional habitats or ancestral territories, and who identify themselves as being part of a distinct cultural group, descended from groups present in the area before modern states were created and current borders defined. They generally maintain cultural and social identities, and social, economic, cultural and political institutions, separate from the mainstream or dominant society or culture.” ([26], para. 1)

Primary health was defined as:

“…socially appropriate, universally accessible, scientifically sound first level care provided by health services and systems with a suitably trained workforce comprised of multi-disciplinary teams supported by integrated referral systems in a way that: gives priority to those most in need and addresses health inequalities; maximises community and individual self-reliance, participation and control; and involves collaboration and partnership with other sectors to promote public health. Comprehensive primary health care includes health promotion, illness prevention, treatment and care of the sick, community development, and advocacy and rehabilitation.” ([27], para. 3)

#### Types of sources

All qualitative, quantitative, economic and mixed methods studies were considered for inclusion in the original scoping review. In addition reviews and systematic literature reviews of programs that meet the inclusion criteria were also retrieved. Only literature published in English from September 1978 were considered in the original scoping review as this is the date that the Declaration of Alma Ata which outlined primary health care was adopted at the International Conference on Primary Health Care. [28]

#### Search terms

Initial search terms used in the original scoping review included Aboriginal, Aborigine, Indigenous, first nation, Maori, Inuit, American Indian, primary health care, comprehensive primary health care, medical service, health service, community care, community health care, model.

#### Search strategy

A three-step search strategy was utilized in the original scoping review. An initial limited search of PubMed and CINAHL was undertaken followed by an analysis of the text words contained in the title and abstract, and of the index terms used to describe the article. Second, a search using all identified keywords and index terms was then undertaken across EBSCO, CINAHL, Embase, Informit, Mednar and Trove. Third, the reference list of all identified reports and articles was searched for additional studies. In addition, academics from universities with expertise in Indigenous health services were contacted and asked to identify literature (particularly grey literature) that meets the review inclusion criteria.

Articles were assessed for inclusion in the initial scoping review by title and abstract. Full text of the articles were retrieved if they meet the inclusion criteria or if further examination was required. Two reviewers independently confirmed that the full text article meet the inclusion criteria. Any disagreements was decided by a third reviewer.

### This framework synthesis

To be considered for this new framework synthesis, papers must have been included in the previous scoping review and present findings related to one or more of the five stages included in Levesque et al. [23] framework (Fig. 1). Findings from these papers were firstly extracted and then imported into NVivo 10. A framework synthesis [29] was used to aid in the analyse and the interpretation of the extracted findings. This entailed identifying a priori framework, in this case the five stages offered by Levesque et al’s framework [23] and then charting and sorting the findings from the included papers into the five stages identified by Levesque and colleagues framework (Table 1).

**Table 1:** A Priori Framework

Stage One: Perceptions of Need and Desire for Health Care	
• Ability to Perceive	
• Approachability	
Stage Two: Health Care Seeking	
• Ability to Seek	
• Acceptability	
Stage Three: Health Care Reaching	
• Ability to Reach	
• Availability and Accommodation	
Stage Four: Health Care Utilisation	
• Ability to Pay	
• Affordability	
Stage Five: Consequences of Accessing Care	
• Ability to Engage	
• Appropriateness	

Findings within each of these five stages were then thematically analysed by one of the authors and an interpretation of the key characteristics developed through a consultative process involving all members of the research team

## Results

Of the 62 papers included in the original scoping review, 50 met the inclusion criteria for this framework synthesis [see Additional file 1: Table S1]. Twenty four of these papers reported on Indigenous Health Care Services based in Australia, 15 within the United States, four in Canada and New Zealand, two in South America and one in Papua New Guinea. The majority of the papers (*n* = 30) included in this synthesis reported on a range of primary health care services. A small number of included papers reported on services which were specifically designed to address maternal and child health (*n* = 5), mental health (*n* = 4), chronic disease management (*n* = 3), oral health (*n* = 3), eye health (*n* = 1) cancer treatment (*n* = 1) and women’s health (*n* = 1). Findings from the synthesis of evidence identified a number of factors related to all five stages of Levesque et al’s framework [23] (Table 2).

**Table 2:** Characteristics related to health care service access and accessibility

**Stage One: Perceptions of Needs and Desire for Health Care**
Patients’ Ability of to Perceive–two papers
Very few studies included identified issues relating to Indigenous peoples’ ability to perceive that health care was needed. Of those that did, perceptions were hampered by a denial that a problem existed, low self-esteem and judgement that was impeded by substance abuse [39]. One other study noted that awareness of services was limited for those Indigenous peoples who were not already accessing services [40].
Services’ Approachability – ten papers
A number of papers identified strategies used by Indigenous health care services to increase the approachability of services.
• Raising awareness about services was achieved by working with patients as well as with the patient’s family and their community as a whole [41–43]. In some instances community representatives were employed to encourage people to utilize services that were available [39], while in others health care providers went into the community to promote and educate people about when and how to seek health care [44].
• Building a positive reputation was believed to be important because it meant that people felt confident in referring others to the service [45, 46]. Services also worked with community members in order to reinforce that their concern was for the health and wellbeing of the community as a whole [47].
• Providing health care within the community helped to raise the profile of services [48], improve health literature and at the same time provided staff with an occasion to opportunistically offer services to people who were in need of assistance but had not yet sought care [39, 49, 50].
**Stage Two: Health Care Seeking**
Patients’ Ability to Seek–eight papers
Prioritizing the needs of others over themselves often prevented Indigenous peoples from seeking health care [39, 51]. The ability to seek care was also limited when culturally appropriate services were not available [39, 47, 49, 52–55] or where concern for maintenance of confidentiality was raised [49].
Services’ Acceptability – 19 papers
The acceptability of services provided for Indigenous peoples was paramount to improving access. In particular this related to providing services that understood and were able to account for the values, beliefs and understandings of the communities they served.
• Providing culturally appropriate care was achieved by seeking out and understanding the cultural values and beliefs of the community [39] including gender specific spaces [44, 54]. It is based on respect, social justice, participation, equality, access, learning and collaboration [50], incorporating for example local language(s), beliefs, gender and kinship systems [47]. Importantly, culturally appropriate care is free from any racism or discrimination [44].
• Employing culturally appropriate staff who understand and respect the cultural values and beliefs of the community was considered to be an important aspect of acceptable health care [56]. While Indigenous health care staff were the preferred option [47], where not available, ensuring that non-Indigenous staff received appropriate cultural training was crucial [8, 57].
• Broadening models of care to encompass a more holistic sense of health including aspects of social, emotional and cultural wellbeing [42, 43, 50, 58] was considered important. In some instances this also involved providing more traditional methods of health care including partnering with traditional healers [41, 44, 49, 56, 59, 60]. A more holistic model also encompassed interventions targeting the social determinants of health including food distribution [44] and housing programs [52, 54].
• Offering a welcoming environment where the community felt comfortable and at ease was important for encouraging community members to access care [44, 61]. This was achieved through the use of cultural artefacts [51], local language [62] and comfortable surroundings [44].
**Stage Three: Health Care Reaching**
Patients’ Ability to Reach–eight papers
Transport was considered to be the main factor which inhibited Indigenous people from reaching services [63, 64] followed by a lack of communication services including telephones [65]. The lack of transport was further exacerbated when health care services were located outside of peoples’ communities [39, 40, 45, 51, 53].
Services’ Availability and Accommodation – 23 papers
Given that many of their patients were often hampered in accessing care either by distance and/or through a lack of transport, Indigenous health care services often went to great lengths to ensure that patients were able to engage with health care.
• Delivering outreach services in a variety of settings [48, 66] including rural, remote [51, 67] and urban [68] communities. Outreach staff attended patients in their own homes [49, 50, 69] and within organisations such as prisons [42]. As well as providing generalist care, outreach services focused on maternal and child health, screening [39, 51], social and emotional wellbeing [41, 70], health promotion programs, and dental care [50, 71]. Outreach services were considered to be a crucial part of a comprehensive model of care [72], particularly for frail aged and disabled Indigenous peoples [54, 67], those had no access to transport [63, 65] and lived significant distances from the health care service [73].
• Providing transport was frequently noted as an effective way of improving availability of services [8, 39, 50, 51, 54, 61, 63]. Where communities were situated in particularly remote areas, transport could extend to the use of small planes to ferry people to and from appointments [51].
• Providing flexible appointments was another way in which some services sought to make health care more available including walkin services [44, 54] and less structured approach to appointments [8]. In some instances this also included extended opening hours [40, 44, 50, 65, 74, 75] and the use of electronic health records which can be reviewed outside by patients and staff outside of scheduled appointments [75, 76].
**Stage Four: Health Care Utilization**
Patients’ Ability to pay–six papers
The cost of health care was a key barrier to accessing health care for Indigenous peoples [53, 64]. In some cases patients could only afford part of the cost while others did not have the means to pay at all [65]. Patients in some cases lived below the poverty line [39]. It was not just the cost of the service that was prohibitive, but also associated costs of travelling to and in some cases remaining at the health care service for extended care [74]. People often made choices based not only on quality but also on cost [40, 55].
Services’ Affordability–14 papers
Providing affordable services often proved difficult for many of the Indigenous health care services included in this review primarily because patients were often unable to afford the true cost of care and funding from other sources was limited.
• Providing cost effective care was considered crucial for ensuring accessibility of health care for Indigenous peoples on low incomes [77]. This often meant reducing charges to many patients [56, 65] or providing free services [44]. In some instances cost effective care also extended to the provision of free medicines [78] and dental care [71], particularly in the case of low income earners [64].
• Managing within constrained budgets was an issue that hampered some services from offering cost effective care. In some instances the cost incurred in providing care was greater than the normal charge to patients [56]. One service reported having to limit some of the more expensive services such as outreach visits due to lack of funding [54]. Services reported receiving insufficient economic support to provide the care that was needed [44, 51, 64, 79, 80].
**Stage Five: Consequences of Accessing Health Care**
Patients’ Ability to Engage–three papers
Engaging with both individual patients and the community more generally took time and patience [46]. Communities that felt a sense of ownership over the service were more likely to engage and then importantly remain engaged with health care services [76]. It was also the case that patients were more comfortable talking about their health with an Indigenous staff member or while participating in cultural activities that some Indigenous services offered to community members [54].
Services’ Appropriateness–17 papers
Many of the Indigenous health care services attempted to engage with the communities they served, and in some cases the service was owned and managed by local Indigenous peoples. Appropriateness of services also related to ensuring a sense of holistic care whereby barriers to accessing any service were reduced.
• Engaging with community to determine their needs was considered crucial to ensuring the acceptability of services [8, 41, 45, 50, 51, 81].
• Ensuring community ownership whereby services that are initiated, planned, governened and managed by the local community was believed to result in the most appropriate cultural models [40, 56, 58, 82].
• Coordinating care meant that patients received a holistic service from a multi-disciplinary team with no internal barriers to access [41, 49, 75]. For some Indigenous peoples with high needs this extended to coordinated home services [62].
• Integrating services improved the ability of the service to meet the holistic needs of the community [44, 56, 72] by ensuring that patients were supported to access care not available within the Indigenous health service [41, 46, 50, 62, 70].

Levesque et al’s [23] accessibility framework was a relatively useful tool for identifying the range of issues which influence access to as well as the ways in which Indigenous health care services address these issues. However, findings suggest two additional improvements, at least in relation to access to health care services for Indigenous peoples. First, the broader health care system rather than a particular service or the user also appeared to influence access to and acceptability of care. Funding was the most obvious system issue identified by this framework synthesis. As indicated in Table 2 above, Indigenous health care services operated within constrained budgets resulting in a reduction of services for Indigenous peoples.

**Fig. 2:**
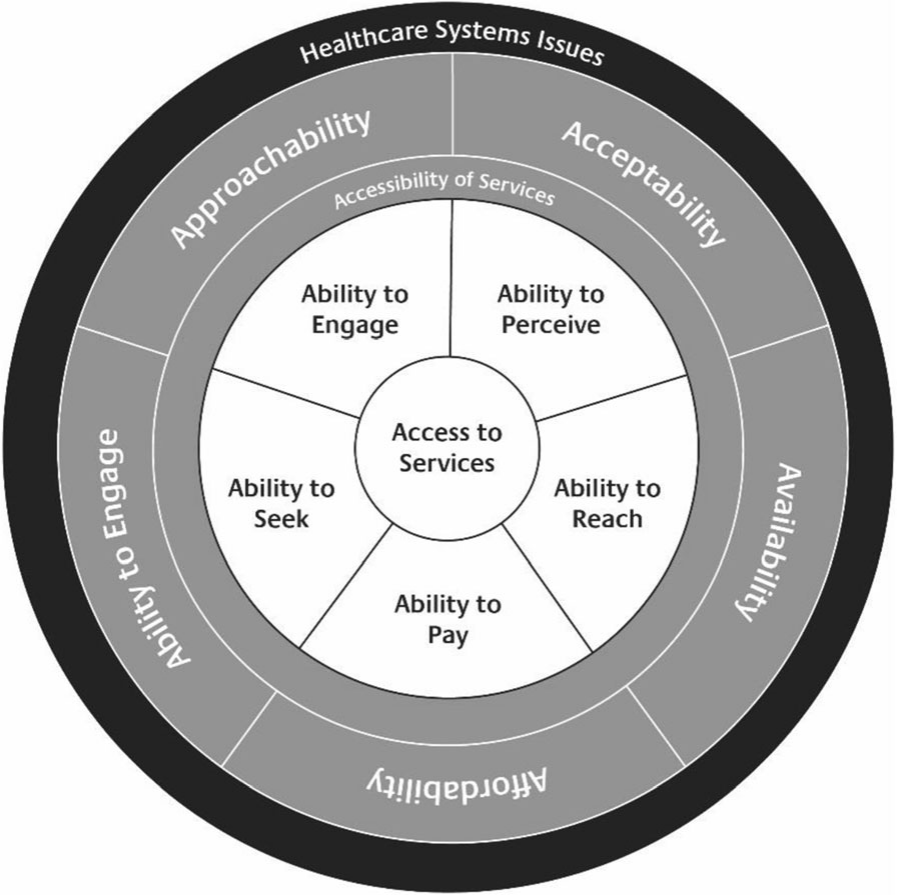
Accessibility Framework for Indigenous peoples accessing Indigenous primary health care services

Our second finding relates to the linearity of Levesque et al’s framework [23] which, in the case of Indigenous health care services did not accurately portray relationships between features of access and accessibility. For example, we found a closer relationship between the ability to pay and the ability to reach than was suggested by the original linear framework. For example, studies demonstrated that the cost of transport to the health care service was as prohibitive as the cost of health care. We also found that the ability to engage was as relevant to health care acceptability as it was to appropriateness. A non-linear representation which provides space for factors relating to health care system systems more broadly (Fig. 2) may better illustrates the many inter-related features that influence access to and acceptability of health care services.

## Discussion

The primary aim of this framework synthesis was to explore access to (factors related to Indigenous patients, their families and communities) and accessibility of (factors relating to services) Indigenous health care services using the Levesque et al. [23] framework. We found issues relating to both the social and cultural determinants of health hampered Indigenous patients’, their families’ and communities’ from accessing care. Poverty was a prominent social determinant of health issue with some Indigenous peoples finding it difficult to afford either transportation to, or the costs of, obtaining services. This review also found that a lack of basic communication infrastructure within communities such as telephones prevented access to health care guidance and advice. To overcome these issues some Indigenous health care services provided transport to and from their facility, or alternatively provided outreach services which delivered care into the patient’s home. Despite limited funding, Indigenous health care services also subsidised these costs for Indigenous peoples on low incomes. While this review highlighted poverty and the lack of communication infrastructure, other related social determinant of health issues such as unemployment and lower levels of education also impact on an Indigenous persons’ ability to access health care services [30]. Health care services that are both cognisant of and able to address the social determinants of health relevance within their particular context will be crucial for improving access to health care services for Indigenous communities.

The types of cultural determinants of health that influenced Indigenous peoples’ ability to access health care related to the ability of the health care service to understand and take account of local beliefs and values when providing care. In this framework synthesis we found that community acceptance was key to both seeking and engaging with health care services. The acceptability of services depends on health care providers understanding the cultural, historical and social fabric of the communities they serve [31]. However, simply understanding is not sufficient. Instead a deeper level of interaction and thoughtful practice that ensures culturally safe services, as defined by those who receive services, is required [32]. To support this view, we found that Indigenous health care staff, particularly those from local communities, were associated with increased Indigenous health care engagement. Indigenous health care services included in this review also actively sought to engage with and learn from local Indigenous peoples. In some instances Indigenous health care services were owned and managed by local Indigenous peoples resulting in a sense of community ownership and promoting the use of culturally safe models of health care.

Indigenous health care services may therefore provide the best opportunity to address access because they are in a better position to address the types of social and cultural determinants of health faced by Indigenous communities. They are, for example, generally situated within or at least near to the communities they serve and are more likely to be aware of local values, norms as well as health care needs. Indigenous health care services are also more willing to work with communities in order to respond to local needs. Importantly, Indigenous services owned and managed by Indigenous peoples are more likely to develop culturally safe models of care [33]. In contrast, mainstream services are generally set up to cater for dominant often non-Indigenous cultures and may not have the resources required to respond to the needs of others. Mainstream services also tend to operate within a set of socially constructed values and norms which can at times, be at odds with Indigenous communities’ beliefs and values – the delivery of mainstream services being predominantly influenced by the biomedical model rather than a more holistic sense of health adopted by many Indigenous peoples [34].

Finally, we suggest two changes to Levesque and colleagues’ framework when evaluating Indigenous peoples’ access to and the accessibility of Indigenous health care services. The first relates to the inclusion of the health care system as part of the framework. Health policies and practices at a systems level have a profound effect on the ability of Indigenous peoples to access health care [35]. While funding was the health care system issue identified in this review, national policies in relation to, for example, the provision of outreach services will also be crucial for improving health outcomes for Indigenous peoples [36]. The second change emanating from this framework synthesis relates to a move from a linear perspective of access and accessibility to one that better represents the inter-connectedness of all of the identified features. Journeys into and through health care services for Indigenous peoples, particularly in relation to those living with chronic illness, are anything but linear. There are often no clearly defined entry or exit points. Instead, an Indigenous patient journey will depend on the individual, their family and geographical context [37]. Common factors for successful navigation include, however, the importance of culturally safe and wherever possible, locally owned Indigenous health care services that are able to understand and meet the needs of the communities they serve [38]

## Limitations

While there were a large number of included papers in this framework synthesis, we acknowledge that this does not capture all of the issues related to access to or accessibility of Indigenous health care services for four reasons. First, no matter how systematic, there is always a possibility that the original scoping review [25] did not identify all potential papers. Second, the aim of the original scoping review was to identify and describe Indigenous service delivery models rather than necessarily Indigenous peoples’ views on access to health care more generally. While access and accessibility were common themes emerging from the scoping review it is likely that other studies focusing on the accessibility of Indigenous health care services could have informed the specific objectives of this synthesis but did not meet the scoping review’s inclusion criteria. Third, while the papers included identified important social and cultural determinants of health issues relating to access, we believe that further research is required in order to increase our understanding of these issues. Finally, we acknowledge a need to further explore factors relating to the health care system which facilitate as well as those that impede access to and the accessibility of Indigenous health care services.

## Conclusion

The framework synthesis outlined in this paper makes a number of contributions to the body of knowledge around access to and accessibility of health care services. In particular, we demonstrate how social and cultural determinants of health influence the extent to which Indigenous peoples are able to access health care services. Importantly, we also describe ways in which Indigenous health care services are addressing these issues and why they are best placed to do so. Based on outcomes from this framework synthesis, we have proposed further additions to Levesque et al’s [23] accessibility framework including the inclusion of systems issues including those relating to the funding of Indigenous health care services, which appear to influence access to and acceptability of health care services.

## Additional file

Additional file 1: Table S1. File contains summary of papers included in the framework synthesis. (PDF 197 kb)

## Abbreviations

NHMRC: National Health and Medical Research Council
