# Supplementary material for: Access to primary health care services for Indigenous peoples: A framework synthesis
Source: Int J Equity Health. 2016 Sep 30;15:163. doi: 10.1186/s12939-016-0450-5 (PMC5045584; doi:10.1186/s12939-016-0450-5)
Supplement: Additional file 1: Table S1. — File contains summary of papers included in the framework synthesis. (PDF 197 kb) [file 12939_2016_450_MOESM1_ESM.pdf]

Additional File 1: Summary of Papers included in the Framework Synthesis

First Author (Year)	Country	Focus of the Service Delivery Model	Region	Study Type
Bennett (1988)	Australia	General	Urban	Case Study
Copeman (1988)	Australia	General	Not Applicable	Opinion
Kahn (1988)	United States	Mental health	Rural	Evaluation
Berner (1992)	Alaska	General	Remote	Case Study
West (1993)	United States	General	Not Applicable	Case Study
Campbell (1995)	Australia	General	Urban	Case Study
Chiarchiaro (1997)	United States	Oral Health	Urban	Qualitative descriptive
Wakerman (1998)	Australia	General	Remote	Case Study
Brant (1999)	United States	Womens Health	mixed	Evaluation
Crengle (2000)	New Zealand	General	Not Applicable	Opinion
Smith (2000)	United States	General	Not Applicable	Evaluation
Baldwin (2001)	Alaska	Maternal and child health	Remote	Qualitative descriptive
Bartlett (2001)	Australia	General	Remote	Case Study
Taylor (2001)	Australia	General	Not Applicable	Opinion
Ediotr (2002b)	Australia	Mental health	Urban	Case Study
Benoit (2003)	Canada	Maternal and child health	Urban	Case Study

Dawson (2003)	Australia	Chronic disease	Rural	Implementation Project
Lantz (2003)	United States	Cancer	Not Applicable	Participatory action research
Maniapoto (2003)	New Zealand	Chronic disease	Not Applicable	Evaluation
Nebelkopf (2003)	United States	General	Urban	Case Study
Lyford (2005)	New Zealand	General	mixed	Case Study
Panaretto (2005)	Australia	Maternal and child health	Rural	Evaluation
Kelaher (2006)	Australia	General	Remote	Evaluation
Allison (2007)	United States	General	Not Applicable	Case Study
Eby (2007)	United States	General	Urban	Case Study
Poroch (2007)	Australia	Prison health	Urban	Qualitative descriptive
Tyree (2007)	United States	General	Rural	Qualitative descriptive
Gabrysch (2009)	South America	Maternal and child health	Rural	Evaluation
Maar (2009)	Canada	Mental health	Rural	Case Study
Stewart (2009)	Australia	General	Rural	Opinion
Birks (2010)	Australia	General	Remote	Case Study
DiGiacomo (2010)	Australia	General	Urban	Evaluation
Auclair (2012)	Canada	Mental health	Rural	Case Study
Davy (2012)	Asia	General	Remote	Implementation Project
Dyson (2012)	Australia	Oral Health	mixed	Evaluation

Murphy (2012)	Australia	Maternal and child health	mixed	Evaluation
Poroch (2012)	Australia	Prison health	Urban	Qualitative descriptive
Arora (2013)	Canada	Eye Health	Remote	Evaluation
Driscoll (2013)	Alaska	General	mixed	Evaluation
Johnston (2013)	Alaska	General	mixed	Evaluation
Freeman (2014a)	Australia	General	Not Applicable	Evaluation
Freeman (2014b)	Australia	General	mixed	Case Study
Gajjar (2014)	Australia	General	Urban	Case Study
Janssen (2014)	New Zealand	Chronic disease	Urban	Evaluation
Langwell (2014)	United States	General	Remote	Evaluation
Panaretto (2014)	Australia	General	mixed	Opinion
Pelcastre-Villafuerte (2014)	South America	General	Rural	Evaluation
Tongs (2014)	Australia	General	Urban	Case Study
Campbell (2015)	Australia	Oral Health	mixed	Case Study
Reeve (2015)	Australia	General	Remote	Evaluation
